# Multisensory spatial perception in visually impaired infants

**DOI:** 10.1016/j.cub.2021.09.011

**Published:** 2021-11-22

**Authors:** Monica Gori, Claudio Campus, Sabrina Signorini, Eleonora Rivara, Andrew J. Bremner

**Affiliations:** 1 Unit for Visually Impaired People, Istituto Italiano di Technologia, 16152 Genova, Italy; 2 Centre of Child Neurophthalmology, IRCCS Mondino Foundation, 27100 Pavia, Italy; 3 Istitute Nursery EdB, 16152 Genova, Italy; 4 School of Psychology, University of Birmingham, Birmingham B15 2SB, UK

**Keywords:** blindness, touch, spatial perception, spatial representation, perceptual development, multisensory development, auditory perception, auditory localization, body representation, visual experience

## Abstract

Congenitally blind infants are not only deprived of visual input but also of visual influences on the intact senses. The important role that vision plays in the early development of multisensory spatial perception^1^, ^2^, ^3^, ^4^, ^5^, ^6^, ^7^ (e.g., in crossmodal calibration^8^, ^9^, ^10^ and in the formation of multisensory spatial representations of the body and the world^1^^,^^2^) raises the possibility that impairments in spatial perception are at the heart of the wide range of difficulties that visually impaired infants show across spatial,^8^, ^9^, ^10^, ^11^, ^12^ motor,^13^, ^14^, ^15^, ^16^, ^17^ and social domains.^8^^,^^18^^,^^19^ But investigations of early development are needed to clarify how visually impaired infants’ spatial hearing and touch support their emerging ability to make sense of their body and the outside world. We compared sighted (S) and severely visually impaired (SVI) infants’ responses to auditory and tactile stimuli presented on their hands. No statistically reliable differences in the direction or latency of responses to auditory stimuli emerged, but significant group differences emerged in responses to tactile and audiotactile stimuli. The visually impaired infants showed attenuated audiotactile spatial integration and interference, weighted more tactile than auditory cues when the two were presented in conflict, and showed a more limited influence of representations of the external layout of the body on tactile spatial perception.^20^ These findings uncover a distinct phenotype of multisensory spatial perception in early postnatal visual deprivation. Importantly, evidence of audiotactile spatial integration in visually impaired infants, albeit to a lesser degree than in sighted infants, signals the potential of multisensory rehabilitation methods in early development.

**Video abstract:**

## Highlights

• Visually impaired infants have a distinct phenotype of audiotactile perception
• Infants with severe visual impairment (SVI) place more weight on tactile locations
• SVI infants show attenuated audiotactile spatial integration and interference
• SVI infants do not show an influence of body representations on tactile space

## Teaser

Gori et al. show that infants with severe visual impairment have a distinct phenotype of multisensory spatial perception. Compared to sighted infants, they show attenuated audiotactile spatial integration and interference, have a more limited influence of body representations on tactile space, and place a greater weight on tactile than auditory cues.

## Results and discussion

The methods used to study perceptual abilities in human infancy are overwhelmingly vision-centric,^21^^,^^22^ but several techniques are now emerging for examining auditory, tactile, and audiotactile localization in human infancy.^3^^,^^4^^,^^23^ Capitalizing on these new techniques, we presented auditory and tactile stimuli (via two audiotactile devices; see STAR Methods) to the hands of ten infants with severe visual impairment (SVI, six males, median = 23.5 months, range = 5–35 months), and ten sighted infants (S, five males, median age = 27.0 months, age range = 8–31 months). Our visually impaired group comprised children with peripheral visual pathologies (e.g., retinal dystrophies or ocular malformation; see Table S2 for clinical details), rather than pathologies of the central visual system (e.g., optic radiation, visual cortex, and visual association cortex). The sighted and visually impaired infants were matched on age and sex.

**Figure 1:**
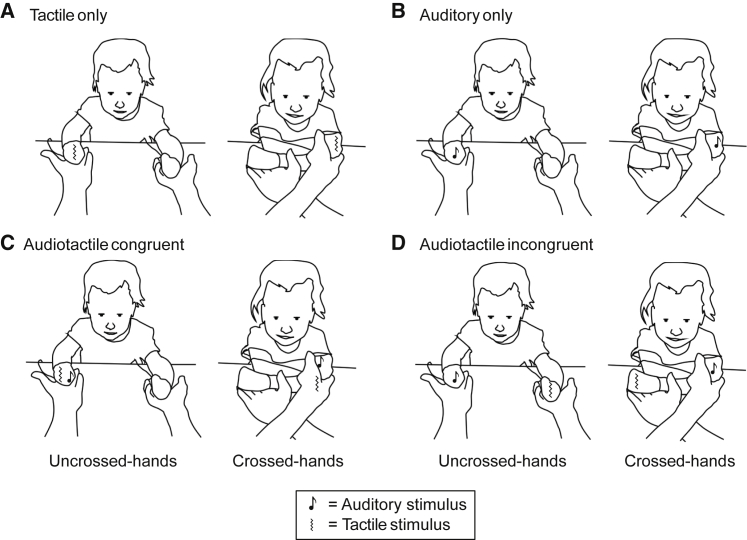
Experimental conditions All panels (A–D) show example stimuli across uncrossed- and crossed-hands posture conditions. The sawtooth lines indicate the vibrotactile stimuli, and the musical notes indicate the auditory stimuli (for more details, see STAR Methods and supplemental information). (A) Tactile-only condition (a vibrotactile stimulus presented to one hand), (B) auditory-only condition (an auditory stimulus presented to one hand), (C) audiotactile-congruent condition (auditory and tactile stimuli presented simultaneously to one hand), and (D) audiotactile-incongruent condition (auditory and tactile stimuli presented simultaneously on different hands).

The four stimulus conditions described in Figure 1 (A–D) were presented in a series of trials. On each trial, a stimulus from one of the four conditions was presented for 1 s, and we recorded movements of the eyes/head and hands/arms made in the following 8 s post-stimulus onset. No task instructions were given to the participants. Responses directed toward or located at one of the two stimulus locations were classified as orienting responses. The orienting responses observed and included in our analyses were movements of the head and eyes toward one of the hands, and movements of one of the hands or one of the arms, including exploratory movements of the fingers and more gross withdrawal movements of the arm; we did not observe any bimanual orienting responses where one hand was moved to a stimulus site on the other). We recorded the numbers and percentages of orienting responses made to the stimulated and unstimulated hands, and the latency (reaction time in seconds, RT) of the orienting responses to the stimulated hand.

The tactile-only and auditory-only conditions provide indications of unisensory localization in these modalities. The audiotactile-congruent and -incongruent conditions allowed us to derive multisensory gain and interference measures by comparison with the tactile-only and auditory-only conditions. The posture manipulations (Figure 1) probe the influence of representations of the body’s typical layout in external space on stimulus localization.^24^, ^25^, ^26^ The sighted and visually impaired groups completed a mean total of 41.1 (SD = 4.2) and 38.7 (SD = 2.9) trials, respectively (see STAR Methods and supplemental information; Table S3 provides details of trials completed per condition).

### Auditory localization

**Figure 2:**
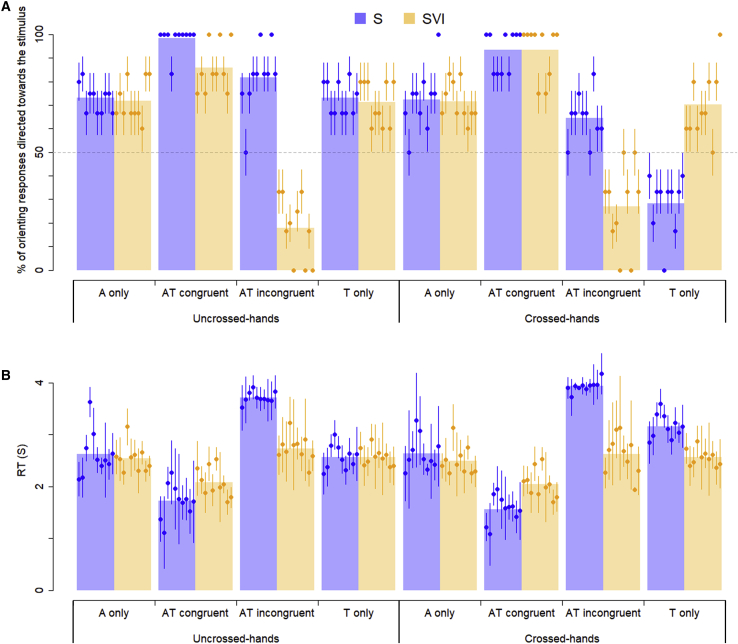
Percentages of orienting responses to the stimulus and mean RTs (A) The percentages of sighted (S) and visually impaired (SVI) infants’ head and manual orienting responses, which were made toward the stimulated hand across stimulus conditions and posture conditions. In the audiotactile-incongruent condition, the data are plotted as the percentage of orienting responses directed to the auditory stimulus. Therefore, in this condition, the percentages of orienting responses directed to the tactile stimulus are 100 minus the percentage values displayed. Small circles and vertical lines respectively represent single participant means and 95% confidence intervals (i.e., ±1.96 SE) ordered with increasing ages (see Table S2). The transparent bars represent the group means. (B) The RTs of responses to the stimulated hand. In the audiotactile-incongruent condition, the reported RTs are of responses to either the tactile or the auditory stimulus. Small circles and vertical lines respectively represent single participant means and 95% confidence intervals (i.e., ±1.96 SE). The transparent bars represent the group means. Only trials with a defined orienting response were included (see also Table S3). See also Figures S1 and S2 and Tables S4 and S5.

A generalized linear mixed-effects model (GLMM), including age in months as a covariate, was fitted to the orienting responses in the auditory-only condition. Orienting response was a binary variable in which “1” coded an orienting response to the stimulated limb (either a hand/arm response with the limb on which the auditory stimulus had been presented, or an eye/head response directed toward the limb on which the auditory stimulus had been presented). “0” coded an orienting response to the unstimulated limb (i.e., a hand/arm response with the other limb or an eye/head response directed toward the other limb). A linear mixed-effects model (LMM) was fitted to the RTs of the orienting responses to the stimulated limb. No significant effects or interactions of group (SVI/S), posture (crossed/uncrossed), or age in months were observed (Table 1; Figure 2).

**Table 1:** Results of the generalized mixed-effects models

	Auditory localization			Tactile localization		Multisensory gain			Crossmodal conflict		
	Stimulus response ∼ group(SVI/S) ^∗^ posture(uncrossed/crossed) ^∗^ age in months + (1|participant)			Stimulus response ∼ group(SVI/S) ^∗^ posture(uncrossed / crossed) ^∗^ age in months + (1|participant)		Stimulus response ∼ group(SVI/S) ^∗^ posture(uncrossed/crossed) ^∗^ condition(tactile only/auditory only/audiotactile congruent) ^∗^ age in months + (1|participant)			Stimulus response ∼ group(SVI/S) ^∗^ posture(uncrossed/crossed) ^∗^ condition(tactile only/auditory only/audiotactile incongruent) ^∗^ age in months + (1|participant)		
Effect	χ^2^(df)	p		χ^2^(df)	p	χ^2^(df)	p		χ^2^(df)	p	
**Generalized linear mixed models (GLMMs) of the orienting responses to the stimulus**											

Condition	–		–			36.922 (2)		<0.001^∗^	15.719 (2)		<0.001^∗^
Group	0.005 (1)		0.945	7.026 (1)	0.008^∗^	2.026 (1)		0.155	2.600 (1)		0.107
Posture	0.044 (1)		0.834	9.036 (1)	0.003^∗^	4.879 (1)		0.027^∗^	6.063 (1)		0.014^∗^
Age (m)	0.436 (1)		0.509	0.067 (1)	0.796	0.003 (1)		0.957	0.013 (1)		0.910
Condition^∗^group	–		–	–	–	7.104 (2)		0.029^∗^	44.419 (2)		<0.001^∗^
Condition^∗^posture	–		–	–	–	6.036 (2)		0.049^∗^	4.988 (2)		0.083
Group^∗^posture	0.035 (1)		0.851	6.350 (1)	0.012^∗^	7.250 (1)		0.007^∗^	9.546 (1)		0.002^∗^
Condition^∗^age (m)	–		–	–	–	0.003 (2)		0.999	0.063 (2)		0.969
Group^∗^age (m)	0.034 (1)		0.855	0.179 (1)	0.672	0.653 (1)		0.613	0.093 (1)		0.760
Posture^∗^age (m)	0.045 (1)		0.832	1.073 (1)	0.300	0.094 (1)		0.760	0.746 (1)		0.388
Condition^∗^group^∗^posture	–		–	–		5.104 (2)		0.078	4.942 (2)		0.085
Condition^∗^group^∗^age (m)	–		–	–		0.273 (2)		0.872	2.064 (2)		0.356
Condition^∗^posture^∗^age (m)	–		–	–		1.412 (2)		0.494	0.466 (2)		0.792
Group^∗^posture^∗^age (m)	0.226 (1)		0.634	0.867 (1)	0.352	0.112 (1)		0.738	0.127 (1)		0.721
Group^∗^posture^∗^condition^∗^age (m)	–		–	–	–	1.361 (2)		0.506	2.237 (2)		0.327

**Linear mixed models (LMMs) of the RTs of orienting responses made to the stimulus**											

Condition	–		–	–	–	982.427 (2)		<0.001^∗^	371.883 (2)		<0.001^∗^
Group	0.089 (1)		0.765	12.697 (1)	<0.001^∗^	0.000 (1)		0.985	28.006 (1)		<0.001^∗^
Posture	0.280 (1)		0.597	17.162 (1)	<0.001^∗^	6.380 (1)		0.012^∗^	13.407 (1)		<0.001^∗^
Age (m)	0.010 (1)		0.922	0.000 (1)	0.986	0.002 (1)		0.966	0.006 (1)		0.940
Condition^∗^group	–		–	–	–	154.177 (2)		<0.001^∗^	218.572 (2)		<0.001^∗^
Condition^∗^posture	–		–	–	–	48.307 (2)		<0.001^∗^	18.606 (2)		<0.001^∗^
Group^∗^posture	0.001 (1)		0.980	53.597 (1)	<0.001^∗^	12.600 (1)		<0.001^∗^	29.063 (1)		<0.001^∗^
Condition^∗^age (m)	–		–	–	–	1.046 (2)		0.593	1.778 (2)		0.411
Group^∗^age (m)	0.947 (1)		0.331	2.116 (1)	0.146	2.562 (1)		0.109	2.451 (1)		0.117
Posture^∗^age (m)	0.286 (1)		0.593	2.433 (1)	0.119	0.082 (1)		0.774	0.073 (1)		0.788
Condition^∗^group^∗^posture	–		–	–	–	40.711 (2)		<0.001^∗^	12.632 (2)		0.002^∗^
Condition^∗^group^∗^age (m)	–		–	–	–	1.800 (2)		0.406	0.935 (2)		0.626
Condition^∗^posture^∗^age (m)	–		–	–	–	0.237 (2)		0.897	0.116 (2)		0.944
Group^∗^posture^∗^age (m)	0.934 (1)		0.334	0.378 (1)	0.538	0.003 (1)		0.403	0.103 (1)		0.748
Group^∗^posture^∗^condition^∗^age (m)	–		–	–	–	0.921 (2)		0.164	0.331 (2)		0.847

Top: GLMMs applied on orienting responses. Bottom: LMMs used on RTs. ^∗^p < 0.05. See also Figures S1 and S2 and Table S1.

The absence of a reliable effect of group on auditory localization suggests that the visually impaired infants’ auditory localization abilities were comparable to those of the sighted infants in speed and accuracy. This result is commensurate with demonstrations that auditory localization does not require visual calibration to develop,^27^ but it conflicts with findings of sound localization deficits in blind children and adolescents.^5^, ^6^, ^7^^,^^21^ There are several potential explanations of this discrepancy. Observations of auditory localization deficits were made in studies where blind children and adolescents were required to localize external auditory objects rather than auditory locations on the body, and it is at least possible that visual impairment has differential effects on representations of bodily and external space. Another possibility is that the visually impaired infants studied here benefitted from the relatively simple testing scenario we used. Indeed, blind adult participants can actually perform better than sighted adults when localization tasks are simple,^28^ lending weight to this explanation.

### Tactile localization and body representations

Sighted adults show crossed limb deficits in tactile localization because they incorrectly assign tactile stimuli to the hand resting on the side of space where the stimulus would typically occur when the limbs are in a canonical posture.^20^^,^^29^ In similar tasks, congenitally blind adults show no such deficit, indicating that this extra-somatosensory influence of body representations on tactile perception develops from visual experience.^2^ Studies of typically developing infants, and the effects of removing congenital cataracts in infancy, show that the critical influence of vision occurs after 4–5 months of age.^1^^,^^15^ We therefore predicted that the sighted infants older than 4 months of age would show a greater crossed-hands deficit than visually impaired infants.

A GLMM, including age in months as a covariate, and group (SVI/S) and posture (crossed/uncrossed) as fixed effects, was fitted to the orienting responses in the tactile-only condition (the same binary code was used as for the GLMM reported above), and a similar LMM was fitted to the RTs of the orienting responses to the stimulated hand. These models revealed effects of group (SVI/S) and posture (crossed/uncrossed), and an interaction of group × posture (Table 1). Post hoc comparisons (see supplemental information) revealed that the sighted infants showed slower and less accurate responses in the crossed-hands condition, but that the visually impaired infants did not (Figure 2), with the interaction driven by groupwise differences in the crossed hands condition. The visually impaired infants matched the best performance of the sighted infants across both postures. Because eye/head orienting necessarily involves an external visual frame of reference, we conducted additional analyses that confirmed the effects and interactions when only the hand/arm responses were included.

Thus, representations of the canonical posture of the body in external space do not influence visually impaired infants’ responses to touch, at least not to the same extent as seen in sighted infants. This finding confirms that vision plays an important role in the influence of body representations on tactile perception during infancy and indicates that visually impaired infants perceive and respond to tactile events differently to how sighted infants do. One explanation for these findings is that visually impaired infants may place greater weight on somatotopic locations, and possibly anatomical features (e.g., whether the limb is the left or right limb, or a hand or foot),^30^ when orienting to bodily locations.

### Multisensory integration

Sighted adults integrate visual, auditory, and tactile cues in order to accurately and efficiently represent and interact with objects and social partners.^31^^,^^32^ Multisensory integration can be measured behaviorally by looking for enhancements in the accuracy and speed of responses under multisensory conditions compared to unisensory conditions (superadditivity^33^^,^^34^). To do this, we fitted a GLMM to the orienting responses in the tactile-only, auditory-only, and audiotactile-congruent conditions (the same binary code was used as for the GLMMs reported above), including age in months as a covariate, and group (SVI/S), posture (crossed/uncrossed), and condition (tactile-only/auditory-only/audiotactile-congruent) as fixed effects. A similar LMM was fit to the RTs of the orienting responses to the stimulated hand. The models revealed effects of posture and condition, and interactions of group × posture, group × condition, posture × condition, and group × posture × condition (The three-way interaction was found in the RT analysis only). Post hoc tests (see supplemental information) indicated that the interactions were driven by the effects in the tactile-only condition already discussed above. Importantly, the effect of condition was driven by faster and more accurate responses in the audiotactile-congruent condition compared to both the tactile-only and auditory-only conditions.

**Figure 3:**
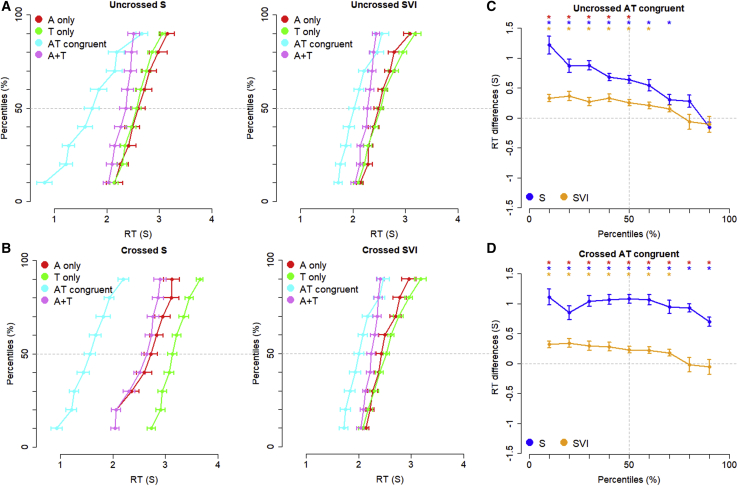
Comparisons of multisensory redundancy gains in severely visually impaired (SVI) and sighted (S) infants. (A and B) Mean cumulative distribution functions (CDFs) of motor reaction times for the sighted and visually impaired infants across uncrossed-hands (A) and crossed-hands (B) postures. For each posture, CDFs are presented for responses to auditory-only (red), tactile-only (green), and audiotactile-congruent (cyan) stimuli, and A+T (violet) and CDF_A+T_ = CDF_A_+CDF_T_. Error bars represent ± SE intervals. (C and D) Tests of redundancy gains (i.e., CDF_A+T_ − CDF_AT_ > 0), are presented for each posture (C: uncrossed; D: crossed), group (blue: sighted infants, S; orange: infants with severe visual impairment, SVI), and centile (10%–90%). Error bars represent ± SE intervals. Blue and yellow asterisks indicate redundancy gains in the S and SVI infants, respectively (p < 0.05 after Bonferroni correction for n = 9 comparisons). Red asterisks indicate significant differences in redundancy gains between S and SVI infants (p < 0.05 after Bonferroni correction for n = 9 comparisons). Only trials with a defined orienting response were included. See also Figure S3.

We next examined RT data across sensory conditions for multisensory redundancy gains (see Figure 3, STAR Methods, and supplemental information). This technique quantifies the reduction in response times due to multisensory integration by comparing RT cumulative distribution functions between the audiotactile-congruent condition and the “race model”^33^ (the sum of the cumulative distribution functions of RTs in the two unisensory conditions, A+T; Figure 3). The race model assumes that if the two sensory channels are independent, the response in the audiotactile-congruent condition will be as fast as the fastest of the response times across the unisensory conditions. Comparing the audiotactile-congruent condition to the race model established that while both groups showed multisensory redundancy gains for both crossed and uncrossed postures in the audiotactile-congruent condition, the gain was stronger in the sighted group (Figure 3; supplemental information).

Previous studies have shown that multisensory integration for audiovisual localization is evident in the first year of life.^35^ Here, we show that both sighted and visually impaired infants integrate spatially redundant tactile and auditory cues to speed their performance rather than processing these cues independently. Since both groups’ gain was observed across both postures, we can conclude that both groups could code relations between auditory and tactile cues in a common spatial frame of reference that was not tied to somatotopic coordinates or the familiar (canonical) positions of the limbs.^36^ Importantly, the visually impaired infants showed less gain overall, indicating that visual experience is required for the typical development of audiotactile spatial integration.

### Resolving crossmodal spatial conflict

Comparing sighted and visually impaired infants’ sensory orienting responses under conditions of audiotactile spatial conflict can provide clues to the relative weightings given to hearing and touch and reveal the nature of crossmodal interactions in spatial perception in visual impairment. A GLMM, including age in months as a covariate, and group (SVI/S), posture (crossed/uncrossed), and condition (tactile-only/auditory-only/audiotactile-incongruent) as fixed factors, was fitted to the infants’ orienting responses. The same binary code was used as for the GLMMs reported above, but in the audiotactile-incongruent condition where stimuli were presented on both hands, an orienting response was coded “1” if the participant responded with or toward the *auditory* stimulus location. A similar LMM was fit to the RTs of the first orienting response made (whether to auditory, tactile, or audiotactile stimuli; see STAR Methods and supplemental information).

The analyses revealed main effects of group (RT analysis only), condition and posture, and interactions of condition × posture (RT analysis only), group × posture, and group × condition. The three-way interaction of group × condition × posture was significant in the RT (LMM) analysis only. Post hoc comparisons revealed that the interactions, including posture, were driven by the effects in the tactile-only condition discussed above. The condition × group interactions were explained by different orienting preferences in the groups: the visually impaired infants showed a significant preference to orient to the tactile stimulus, whereas the sighted infants preferred the auditory stimulus. Furthermore, only the sighted infants showed a significant slowing in the incongruent audiotactile condition compared to the unisensory conditions (further analyses in the supplemental information).

Thus, when a touch is presented on the one hand and a sound on the other, visually impaired infants tended to orient toward the touch and sighted infants to the sound. Interestingly, we only observed crossmodal interference in making an orienting response in sighted infants. The visually impaired infants’ responses (primarily to the touches) were not disturbed by the concurrent presence of a sound on the other hand. Reduced distractor effects are also seen in blind adults across both multisensory and unisensory perceptual tasks.^37^ Our findings indicate that enhanced crossmodal selective attention skills that blind adults show in multisensory tasks may have their origin in infancy. It is also possible that early behavioral patterns of preference and performance that we have observed in visually impaired infants may be a developmental precursor to a more general selective attention advantage in blind adults, even in unisensory tasks. For instance, the selection of tactile in preference to auditory stimuli in early infancy may lead to the attenuated integration of auditory and tactile space reported above. This developmental process could in turn limit the extent to which visually impaired infants/children learn to integrate information across spatial arrays in multisensory space, leading to downstream advantages in selective spatial attention both crossmodally and within individual senses.

### The sensory phenotype of visual impairment in infancy

Congenital visual deprivation has influences far beyond vision itself and means that visually impaired individuals grow up with qualitatively different and, in some circumstances, impoverished spatial representations in their intact senses.^38^ Here, we show how the absence of visuospatial experience affects development in the first years of life. We have found that visually impaired infants, compared to their sighted peers, show (1) faster and more accurate localization of tactile stimuli on the hands when their hands were crossed, suggesting prioritization of the anatomical location of touches and relative neglect of the canonical layout of the body; (2) significantly reduced audiotactile gain in orienting accuracy and response speed, indicating an attenuated spatial integration of auditory and tactile stimuli; (3) greater weight given to tactile orienting under conditions of audiotactile spatial conflict; and (4) reduced interference under audiotactile spatial conflict. We found no statistically reliable differences in auditory localization between sighted and visually impaired infants.

How do these various features of the sensory phenotype of visual impairment arise developmentally? We speculate that, in typical development, the rich input that vision provides concerning both the world close at hand and the world at a distance provides a sensory spatial framework that greatly impacts how auditotactile perception develops. The absence of this rich visuospatial framework can explain several aspects of the phenotype observed here in visually impaired infants: (1) attenuated multisensory spatial integration may arise from the absence of a common visuospatial framework within which to integrate auditory and tactile cues, (2) greater emphasis on anatomical or somatotopic coordinates in touch localization may arise from the absence of visuospatial input concerning the body, and (3) a greater weighting placed on tactile over auditory spatial locations may arise from there being, without vision, a more limited sensory framework within which to orient to the world at a distance. There is also the possibility that some of the features of spatial perception in visual deprivation resulted indirectly from multisensory differences rather than directly from the absence of vision. We have already argued that attenuated multisensory integration of auditory and tactile space might limit interference from auditory distractors. There is also the possibility that the bias to respond to tactile compared to auditory stimuli might also lead both to the attenuated multisensory integration of auditory and tactile space and to the greater weight placed on somatotopic/anatomical coordinates and features.^30^ Indeed, other perceptual differences in visual impairment not studied here may also play a role. For instance, if visual deprivation affects the perceived timing or spatial position of auditory or tactile stimuli,^39^ this might reduce redundancy gains. Longitudinal studies with infant participants are needed to generate a full causal model^40^ describing how visual impairment affects the early development of the sensory phenotype of visual impairment.

The wide-ranging sensory differences we have observed in visually impaired infants help explain some of the behavioral features of blind and visually impaired infants and may also bear important implications for developing early intervention techniques. Visually impaired and blind infants do not reach for objects that produce sounds until the end of the first year, while sighted infants start around 5–6 months.^41^^,^^42^ The greater weight given to the tactile modality may explain the impairments observed in many exploratory behaviors they show. However, the comparable performance of the visually impaired infants in auditory localization shows that proximal auditory spatial cues on the body are readily utilized in early life. It may be possible to build on this to encourage greater exploration of extra-bodily auditory space. We also found that visually impaired infants could integrate auditory and tactile cues, even when the tactile and auditory cues were presented in a conflict between external and anatomical locations (i.e., in the crossed-hands posture). These findings are encouraging in that they show that multisensory integration is available as a tool to shape the functioning of the intact senses in early life.^12^ Such approaches may help visually impaired infants make better use of auditory and multisensory space to help them link the tactile world of the body to the outside world of objects and people.

## STAR★Methods

### Key resources table

**Table:** 

REAGENT or RESOURCE	SOURCE	IDENTIFIER
**Deposited data**		

Experimental data	Zenodo	https://zenodo.org/record/5355402
**Software and algorithms**		

lme4	The Comprehensive R Archive Network (CRAN)	https://cran.r-project.org/web/packages/lme4/
emmeans	CRAN	https://cran.r-project.org/web/packages/emmeans/
car	CRAN	https://cran.r-project.org/web/packages/car/
R 4.0.5	The R Foundation for Statistical Computing Platform	https://www.r-project.org/

### Resource availability

#### Lead contact

Further information and requests for resources should be directed to and will be fulfilled by the lead contact, Monica Gori (Monica.Gori@iit.it).

#### Materials availability

This study did not generate any new unique materials.

### Experimental model and subject details

#### Participants

Ten sighted infants (5 males, median age = 27.0 months, age range = 8-31 months) and 10 visually impaired infants (6 males, median = 23.5 months, range = 5-35 months) took part in this study. Five additional participants (2 sighted and 3 visually impaired infants) were tested, but their data were not included because they completed fewer than 10 trials. Informed consent was obtained from the infants’ parents before commencing the study. The testing took place only if the infant was awake and in an alert and content state. Ethical approval was gained from the Ethics Committee of ASL 3 Genova and from the Ethics Committee of Pavia Area, Fondazione IRCCS Policlinico San Matteo, Pavia (Italy).

#### Clinical details of the visually impaired participants

Full clinical details are provided in Table S2 (Supplemental information). Grating acuity was measured with Teller acuity cards (as is appropriate to pre-verbal children and infants). The Teller acuity cards are a widely-used standardized test for measuring visual acuity in infants and young children that exploits the natural preference of infants to look toward patterns rather than a blank, homogeneous area. During the procedure, the infant is either held or seated alone at the correct distance from the acuity cards, facing the tester. The tester attracts the infant’s attention and when they look straight ahead a card is held up. The cards display a grating on one side and a homogeneous area on the other. Based on a variety of the infant’s cues including fixation, eye movements, pointing, touching, and/or verbalization, the tester makes a decision as to whether the infant can see the grating. Coarser or finer gratings are then presented and repeated until the tester can select the finest grating that the infant can see, which then provides an indication of their acuity.

In general, it is difficult to evaluate the exact visual abilities of such young participants. Severely Visually Impaired participants have, in the best case, minimal visual perceptual abilities at very short distances. For example, at close proximity, they may be able to recognizing the gratings used in the Teller acuity cards or may be able to discriminate the objects of very different dimensions on the basis of their raw contours. In other cases there may be only sporadic perception of lightness in different/sparse parts of the visual field (e.g., due to missing areas of the retina, there may be an extended part of the visual field in which they are totally blind). However, a precise evaluation of the visual field is practically impossible when participants are of the ages studied here.

None of the participants had a history of prenatal infections, fetal distress during delivery, any other perinatal distress, or any metabolic disorders. None of the participants demonstrated any cognitive impairment, and all had good general health status and were classified as normal in a clinical neurological examination. None of the participants were epileptic. All participants demonstrated a normal psychomotor development level based on a clinical evaluation and on the Reynell-Zinkin Scale which is specifically developed for visually impaired infants aged 0-4 years. Cerebral visual impairment was excluded based on anamnesis, clinical and instrumental visual function assessment and neurological examination. Only one SVI participant was born pre-term, a female participant of 29 months of ages. Given that this participant has passed the age at which preterm infants are typically seen and expected to have caught up developmentally (24 months) we did not make any age correction. Moreover, this participant showed patterns of response direction and latency which are in line with the SVI group average.

### Method details

#### Design

Four different stimulus conditions (Tactile only trials, Auditory only trials, Audiotactile congruent trials, and Audiotactile incongruent trials, see Figure 1) were presented across 6 blocks of trials. The first set of 3 blocks and the last set of 3 blocks were identical except that the first 3 were presented to infants with their hands uncrossed and the last 3 blocks were presented with the infants’ hands crossed. Auditory only, Audiotactile congruent and Audiotactile incongruent trials were presented interleaved in blocks 1, 2, 4, and 5 according to a pseudorandom order shown in Table S1, with the only constraint being that the same hand was not stimulated with the same sensory cue (auditory/tactile) on more than 2 consecutive trials. Tactile only trials were presented separately in blocks 3 and 6 (also in a pseudorandomized order, with the only constraint being that the same hand was not stimulated on more than 2 consecutive trials). Each stimulus condition was presented on a maximum of 12 trials in total (6 in each posture), and therefore a maximum of 48 trials was presented in total (24 in each posture). The order of blocks and trials was kept constant across participants.

#### Apparatus and materials

The child was seated on the lap of a parent, who was seated on a chair. A digital video camera located 80 cm in front of the chair, facing the infant’s frontal midline, recorded the movements of the participant’s body (e.g., movements of the hands, arms and head). Video data were recorded at 25 frames per second for offline coding. The auditory and vibrotactile stimuli were delivered by two custom-built serial-controlled audiotactile stimulators^43^ that the experimenter placed in the infant’s palms, securing them in place with a cohesive bandage. The vibrotactile stimulus was a continuous pure tone (112 Hz). The sound was a 926 Hz pure tone pulsed at a frequency of 5 Hz. Each stimulus event lasted for 1000 ms, followed by 8000 ms to allow sufficient time for the infant to react to the stimulus. Stimulus-linked signals were sent to a serial-controlled visual stimulator^43^ placed far behind the infant and out of their view to signal the onset and offset of the auditory and vibrotactile stimuli to the behavioral coder via the video recording. This enabled the infants’ behavior to be observed and coded in a stimulus-locked manner. This visual signal did not indicate which hand the stimulus was presented to in order to ensure that the behavioral coders were unaware of stimulus location. We verified that the pulsed auditory tones delivered to speakers on the hands did not have any tactile effect: in a pilot detection experiment with 3 child participants, we presented an auditory stimulus on one hand over a set of trials. Headphones playing with white noise prevented the children from hearing the sounds. Orientation responses toward the stimulated hand occurred at chance level, demonstrating that orienting responses toward auditory stimuli in the main experiment were not elicited by tactile stimulation arising from the speakers.

#### Procedure

The experiment was conducted by 2 operators, E1 and E2. E1 faced the infant participant, interacted with them, and manipulated their arms gently into crossed and uncrossed postures. E2 observed and triggered the stimuli once the infant was in the correct posture via a MATLAB program. For each trial, E1 played a game with the infant “bouncing” their hands into the correct position and saying “*1,2,3, bù*.” The hands were placed in the allocated posture just before “*bù*,” and the “*bù*” also functioned as a cue for E2 to trigger a stimulus. After each trial, the operators marked it as good or bad. If the trial was marked as bad (e.g., if the infant’s hand moved their hand out of position before the stimulus was delivered), it was repeated (an average of 3 trials per participant were repeated, SD = 2.0). Following the delivery of the stimulus, E1 gently held the infant’s arms in the assigned posture until the infant either moved their hands, or 8000 ms had elapsed, at which point the trial was terminated. In the 8000 ms period following each stimulus, E1 oriented their face to the floor in order not to distract the infant. If the infant became fussy, they were entertained with musical toys until they were sufficiently settled to continue with the study. The study continued for as long as the infant and parent were willing to cooperate or until all trials had been completed.

#### Observational coding

The direction, latency, and type of the infants’ first orienting responses to the stimuli (auditory, tactile and audiotactile trials) on each trial were coded from the video records by two raters naive to the purpose of the study. Both of the raters were also unaware of the side of stimulus presentation but were provided with stimulus onset and offset information. The following coding scheme was used, based on previous approaches.^3^^,^^44^ During the 8000 ms period following the stimulus on each trial, lateral eye movements (saccades), lateral head movements, and unilateral hand/arm movements were all coded as orienting responses. Where infants made bilateral, symmetrical arm movements, these were coded as null responses and the trial was terminated. The hand/arm movements which were coded as orienting responses, included fine movements (i.e., flexions and extensions of the elbow, wrist and finger joints) and gross withdrawal movements (the arm and hand-pulled back toward the infant’s trunk). Bilateral coordination responses (i.e., where the contralateral hand moved toward the other hand), were a coding category, but no such movements were observed in either group.

### Quantification and statistical analysis

#### Inter-observer reliability

The ratings provided by the two independent observers were compared and examined for agreement using Cohen’s Kappa^45^ and weighted Kappa,^46^ which yields an estimate of agreement between two sets of nominal scores. The agreement was “almost perfect.”^47^ Unweighted and weighted Kappa agreements for response side (Left versus Right versus Null) were 0.933 (95% CIs: 0.928-.943) and 0.938 (95% CIs: 0.923-.947), respectively. Unweighted and weighted Kappa agreements for response modality (Arm/hand versus Eye/head versus Null) were 0.946 (95% CIs: 0.939-.953) and 0.942 (95% CIs: 0.925-.962), respectively. In order to optimize the statistical power of the data submitted to analysis, the raters first attempted to come to an agreement on trials where there were divergent ratings for the side and modality of response. This yielded improved reliability across raters. Unweighted and weighted Kappa agreements for response side (Left versus Right versus Null) were 0.975 (95% CIs: 0.962-.989) and 0.983 (95% CIs: 0.983-.983), respectively. Unweighted and weighted Kappa agreements for response modality (Arm/hand versus Eye/head versus Null) were 0.988 (95% CIs: 0.979-.998) and 0.995 (95% CIs: 0.995-.995), respectively.

Following agreement of the response direction and response modality ratings described above, the observers’ ratings for the RTs of the agreed orienting responses were compared. The ratings of the RTs of orienting responses across the two observers were highly correlated, *r*(798) = 0.963, p = < .001 (95% CIs = 0.957-.967). The average disagreement in RT ratings between the coders was computed as the mean of the signed difference between RT estimated by rater 1 and by rater 2, corresponding to 0.04 s (95% CIs = -.32-+.30 s), in other words 1 video frame (video was recorded at 25 frames per second).

#### Description of data and measures

The sighted infants completed a mean of 45.5 trials (SD = 3.0) from a maximum of 48 trials (in total, the group completed 455 trials from a maximum of 480 trials). The visually impaired infants completed a mean of 44.9 trials (SD = 3.8) from a maximum of 48 trials (in total, the group completed 449 trials from a maximum of 480 trials). Trials on which the infant did not demonstrate an orienting response or where the observers could not agree on whether there had been a direction orienting response or in which direction were designated as null trials and excluded from further analysis.

The sighted infants contributed a mean of 41.1 trials (SD = 4.2) in which they made an orienting response (in total, the group contributed 411 trials from a maximum of 455 trials completed). The visually impaired infants contributed a mean of 38.7 trials (SD = 2.9) in which they made a directional orienting response (in total, the group contributed 387 trials from a maximum of 449 trials completed). The mean difference between the numbers of trials contributed by sighted and visually impaired groups was non-significant, t(17.240) = 1.235, p = 0.234.

Arm/hand responses represent the majority of responses in both the sighted group (M = 60.8%; SE = 2.5) and the visually impaired group (M = 61.9%; SE = 2.0). Eye/head responses accounted for 20.5% (SE = 1.7) of orienting responses in the sighted group, and 17.5% (SE = 1.8) of responses in the visually impaired group. Also, both groups contributed a number of trials in which they oriented simultaneously with both eye/head and hand/arm (S mean = 2.3%, SE = 0.8; SVI mean = 6.1%, SE = 0.8) and trials in which they did not show an orientation response (S mean = 5.1%, SE = 3.2; SVI mean = 5.3%, SE = 3.0). The number of trials on which infants contributed orienting responses of different kinds also did not differ between groups: arm/hand responses, t(17.667) = 0.122, p = . 904; eye/head responses, t(17.810) = 1.363, p = 0.190; simultaneous eye/head and hand/arm responses, t(16.873) = 0.941, p = 0.360.

It is important to note that, commensurate with previous findings using similar stimulus presentations in these age groups,^3^^,^^44^ the RTs of the infants’ orienting responses to stimuli presented on their hands (Figure 2) were longer than would be expected in response to the kinds of stimuli typically presented to these age groups – auditory or visual targets usually in extrapersonal space (e.g., Neil and colleagues,^35^ find infant group mean saccadic response latencies ranging from 278 to 1078 ms). Given the longer reaction times observed here, we recorded all responses within 8 s of stimulus onset. However, given the possible spurious effects arising from long latency responses, all analyses were conducted excluding responses with RTs longer than 4 s (3.635% and 2.834% of contributed trials in sighted and visually impaired infants, respectively, with no difference comparing the number of such trials between groups t(17.5) = 1.022, p = 0.32). The analyses excluding responses that occurred after 4 s latency (see Figure S5) provided consistent results with the analyses of all responses reported in the main manuscript.

We calculated percentages of the infants’ orienting responses which were directed to the stimuli in all groups and conditions (see Figure 2). In our analyses of responses in Tactile only trials we were particularly interested in the effect of a non-anatomical frame of reference on orienting responses. And because eye/head orienting involves an external frame of reference by necessity, we conducted additional analyses to confirm effects and interactions when only the hand/arm responses were included (and eye/head responses excluded). In the Audiotactile incongruent condition, because auditory and tactile stimuli were presented on separate hands, we used the auditory stimulus as a reference and calculated the percentage of orienting responses directed to the auditory stimulus (though note that because all other responses were to the tactile stimulus, the percentage responses to the tactile stimulus in the Audiotactile incongruent condition are 100% minus the percentage of responses to the auditory stimulus).

#### (Generalized) linear mixed model analyses

Only trials with an agreement between raters and with a defined orienting response were considered in inferential statistical analyses. All analyses were conducted using R.^48^ Results were deemed significant when p < .05. Where multiple comparisons were used to follow up significant interactions, a Bonferroni correction was applied.

The direction of infants’ orienting responses was expressed as a binary variable for each trial on which and orienting response occurred. “1” coded an orienting response to the stimulated limb (either a hand/arm response with the limb on which the auditory stimulus had been presented, or an eye/head response directed toward the limb on which the auditory stimulus had been presented). “0” coded an orienting response to the unstimulated limb (i.e., a hand/arm response with the other limb or an eye/head response directed toward the other limb). We applied a series of generalized linear mixed models (GLMMs) to these data using a logistic link function. The model fitting was undertaken using the *glmer* function of the lme4 package.^49^ For the latency analyses, a series of linear mixed models (LMMs) were fitted to the RTs of trials where the infants oriented to the hand in which the stimulus was delivered (in the Audiotactile incongruent condition, this was either hand). The model fitting was undertaken using the *lmer* function of the lme4 package. For the GLMMs and the LMMs predictors were evaluated using Type III Wald χ^2^ tests as implemented in the *Anova* function of the car package.^50^ We included random intercepts in all models, allowing for individual differences in baseline performance, but did not have enough power to estimate participant-specific slopes.

Effects and interactions of Posture, Stimulus condition, Group, and Age (in months) were evaluated in the GLMMs and LMMs. Significant fixed effects were further investigated with the *emmeans* function of the emmeans package^51^ by obtaining estimated marginal means (EMMs) and computing their contrasts. Effect sizes were estimated using the *eff_size* function of the emmeans package, using the sigma and the edf estimated by every single model.

The following models were fitted to the orienting responses and latency to investigate: i) Auditory localization, ii) Tactile localization, iii) Multisensory gain, and iv) Crossmodal conflict. Note that in the analysis of tactile localization only hand/arm responses were included. According to Wilkinson’s notation,^52^ the models fitted, were:

i) *Auditory localization* (Auditory only condition): Stimulus response/RT ∼Group(SVI/S)^∗^Posture(Uncrossed/Crossed)^∗^Age in months + (1|participant);
ii) *Tactile localization* (Tactile only condition): Stimulus response/RT ∼Group(SVI/S)^∗^Posture(Uncrossed/Crossed)^∗^Age in months + (1|participant). For 2 way interactions, the correction was applied against all meaningful contrasts, i.e., with each level of one factor, pairwise comparisons were drawn between for each level of the other factor. So when considering the Group x Posture interaction, for each group we compared postures (in emmeans: list(pairwise∼posture|group), n = 2 comparisons), and for each posture we compared groups (in emmeans: list(pairwise∼group|posture), n = 2 comparisons);
iii) *Multisensory gain* (Tactile only, Auditory only, and Audiotactile congruent conditions): Stimulus response/RT ∼Group(SVI/S)^∗^ Posture(Uncrossed/Crossed)^∗^Condition(Tactile only/Auditory only/Audiotactile congruent)^∗^Age in months + (1|participant). For the 3 way Group x Posture x Condition interaction, the correction was applied against all meaningful contrasts, i.e., within each level of two factors, the pairwise comparisons were drawn between all levels of the third. So for each group and condition we compared postures (in emmeans: list(pairwise∼posture|group^∗^condition), n = 6 comparisons), for each condition and posture we compared groups (in emmeans: list(pairwise∼group |condition^∗^posture), n = 6 comparisons), and for each posture and group we compared conditions (in emmeans: list(pairwise∼condition|posture^∗^group), n = 12 comparisons).
iv) *Crossmodal spatial conflict* (Tactile only, Auditory only, and Audiotactile incongruent conditions): Stimulus response/RT ∼Group(SVI/S)^∗^Posture(Uncrossed/Crossed)^∗^Condition(Tactile only/Auditory only/Audiotactile incongruent)^∗^Age in months + (1|participant). For the 3 way Group x Posture x Condition interaction, the correction was applied against all meaningful contrasts, i.e., within each level of two factors, the pairwise comparisons were drawn between all levels of the third. So, for each group and condition we compared postures (in emmeans: list(pairwise∼posture|group^∗^condition), n = 6 comparisons), for each condition and posture we compared groups (in emmeans: list(pairwise∼group|condition^∗^posture), n = 6 comparisons), and for each posture and group we compared conditions (in emmeans: list(pairwise∼condition|posture^∗^group), n = 12 comparisons).

Due to limitations in the data which can be gathered from these participant groups (particularly the number of trials that will be tolerated by infants of these ages), we were able to gather fewer trials per condition than would normally be optimal for fitting of the linear mixed models which we have used here. It is therefore important for the reader to take care in interpreting the findings of these models, particularly the absence of effects. We have correspondingly placed clear qualifications on our interpretation of the absence of effects throughout this article.

#### Race model analyses

A multisensory gain in participants’ responses to bimodal stimuli (compared to the unimodal stimuli) can be determined by examining whether RTs in the bimodal condition are faster than predicted by the race model, in which responses arise from the independent processing of the unisensory signals.^33^

As undertaken by^34^ we compared performance in the Audiotactile condition to the upper bound predicted by the race model. This was done by comparing RTs for a range of deciles (10, 20, 30, 40, 50, 60, 70, 80 and 90%) of the cumulative distribution functions (CDFs) for the Audiotactile condition and the race model (the race model’s CDF is the sum of the Auditory only and tactile only CDFs). We then compared the difference in RTs between the audiotactile CDF and the race model CDF (i.e., multisensory gain) for each decile against zero using one-sample one-tailed t tests (p values were corrected for nine tests for each group using the Bonferroni method). We then performed two-tails t tests to compare the multisensory gains of sighted and visually impaired groups for each decile (p values were corrected for nine tests for each group using the Bonferroni method).

Data were analyzed at group level and when a reaction time bin comprised multiple values from the same participant, the within-participant average was considered. Welch t tests across groups were used as we did not assume variance homogeneity across groups. Specifically, t tests were performed using the t.tets function of R, which automatically corrects degrees of freedom to account for possible non-homogeneous variances between compared samples.

## Data Availability

The datasets generated during this study are available on Zenodo at: https://zenodo.org/record/5355402

## Supplemental information

Document S1. Figures S1–S3 and Tables S1–S5

Document S2. Article plus supplemental information

Video abstract
