# Supplementary material for: Multisensory spatial perception in visually impaired infants
Source: Curr Biol. 2021 Nov 22;31(22):5093–5101.e5. doi: 10.1016/j.cub.2021.09.011 (PMC8612739; doi:10.1016/j.cub.2021.09.011)
Supplement: Document S1. Figures S1–S3 and Tables S1–S5 [file mmc1.pdf]

Current Biology, Volume *31*

Supplemental Information

Multisensory spatial perception

in visually impaired infants

Monica Gori, Claudio Campus, Sabrina Signorini, Eleonora Rivara, and Andrew J. Bremner

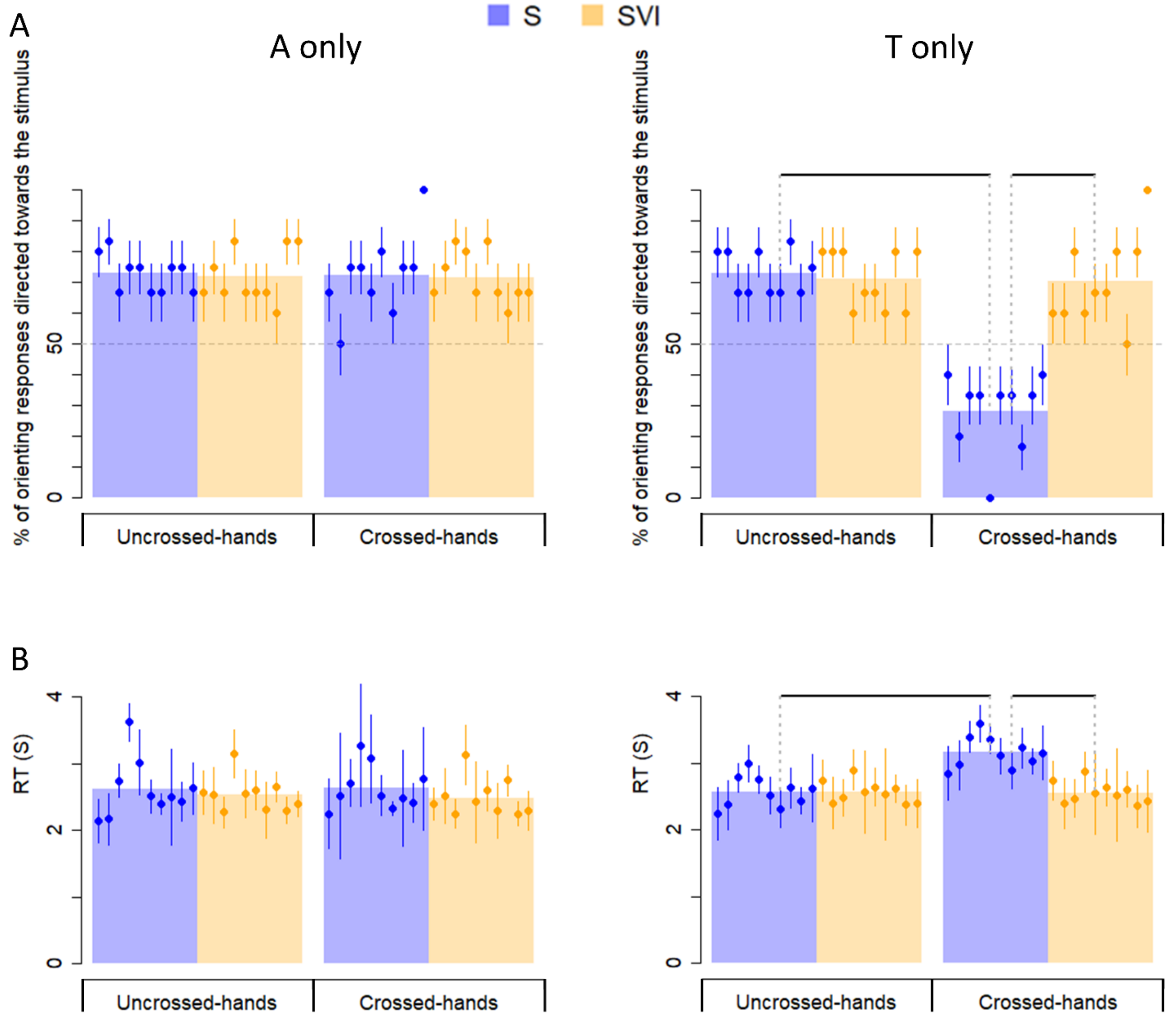

Figure S1. Auditory and tactile localisation. Related to Figure 2 and Table 1. The Auditory only (left) and the Tactile only (right) conditions were considered separately. (A) The percentages of infants' head and manual orienting responses were made towards the stimulated hand across stimulus conditions, posture conditions and groups (S = Sighted infants; SVI = Infants with Severe Visual Impairment). (B) The RTs of responses to the stimulated hand. Small circles and vertical lines represent single subject means and 95% confidence intervals i.e., +/-1.96 SE. Single subject data points are ordered left to right with increasing age in months. Transparent bars represent the group means. Black horizontal lines represent significant post hoc comparisons ($p<.05$) following significant 2 way interactions.

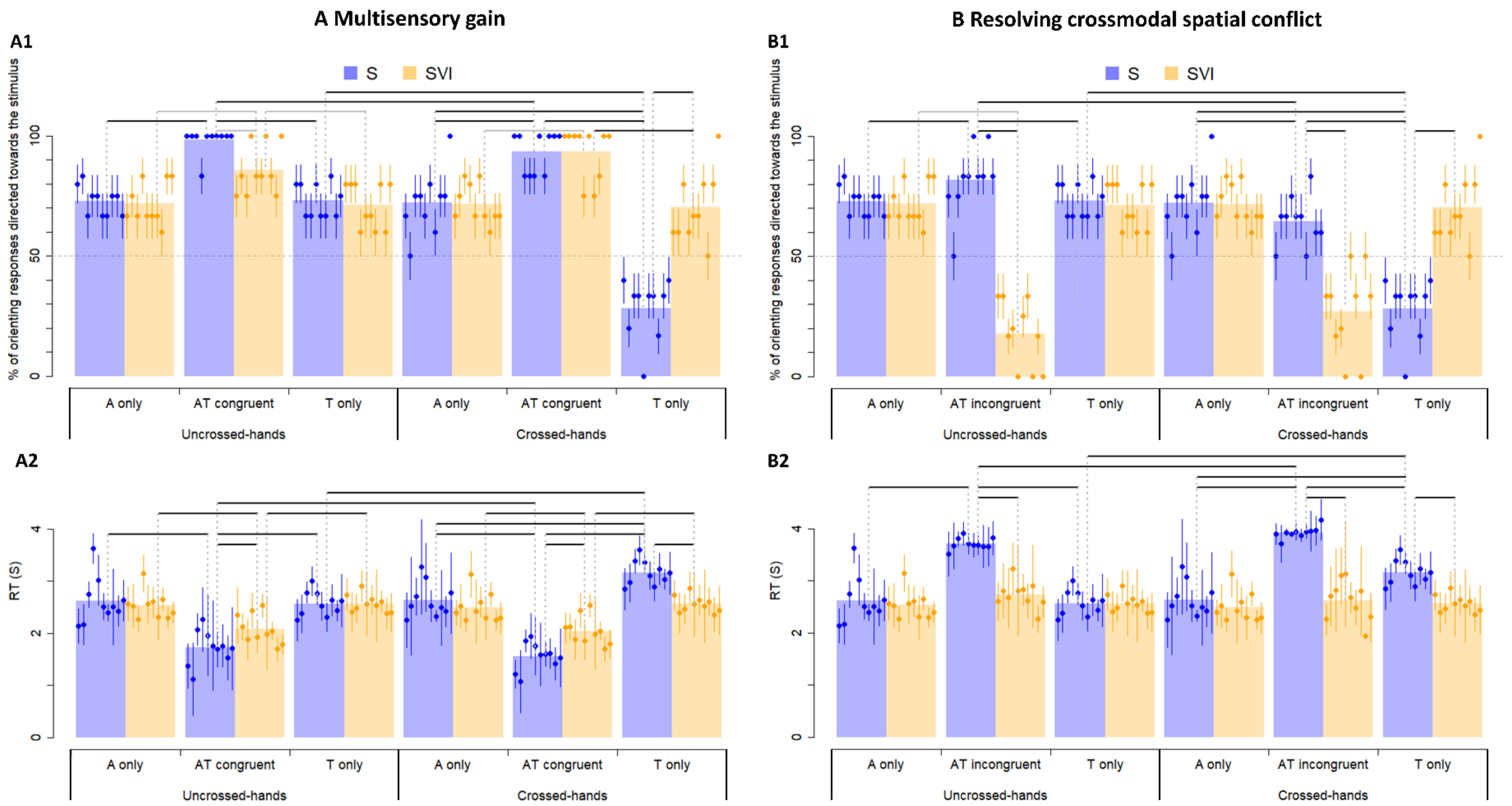

Figure S2. Audiotactile spatial integration and interference. Related to Figure 2 and Table 1. Investigations of multisensory integration (A) and crossmodal conflict (B) via comparisons of infants' head and manual orienting responses made towards the stimulated hand (A1 and B1) and orienting response reaction times (A2 and B2) across stimulus conditions, postures and groups (S = Sighted infants; SVI = Infants with Severe Visual Impairment). In the audiotactile incongruent condition, the data is plotted as the percentage of orienting responses directed to the auditory stimulus. Therefore, the percentage of orienting responses directed to the tactile stimulus is 100 minus the percentage values displayed in this condition. Small circles and vertical lines represent single subject means and 95% confidence intervals i.e., +/-1.96 SE. Single subject data points are ordered left to right with increasing age in months. Transparent bars represent the group means. Post hoc comparisons performed to explore the 3 way interactions are shown by black horizontal lines for significant comparisons ($p<.05$) while grey horizontal lines signal trends ($p<.09$).

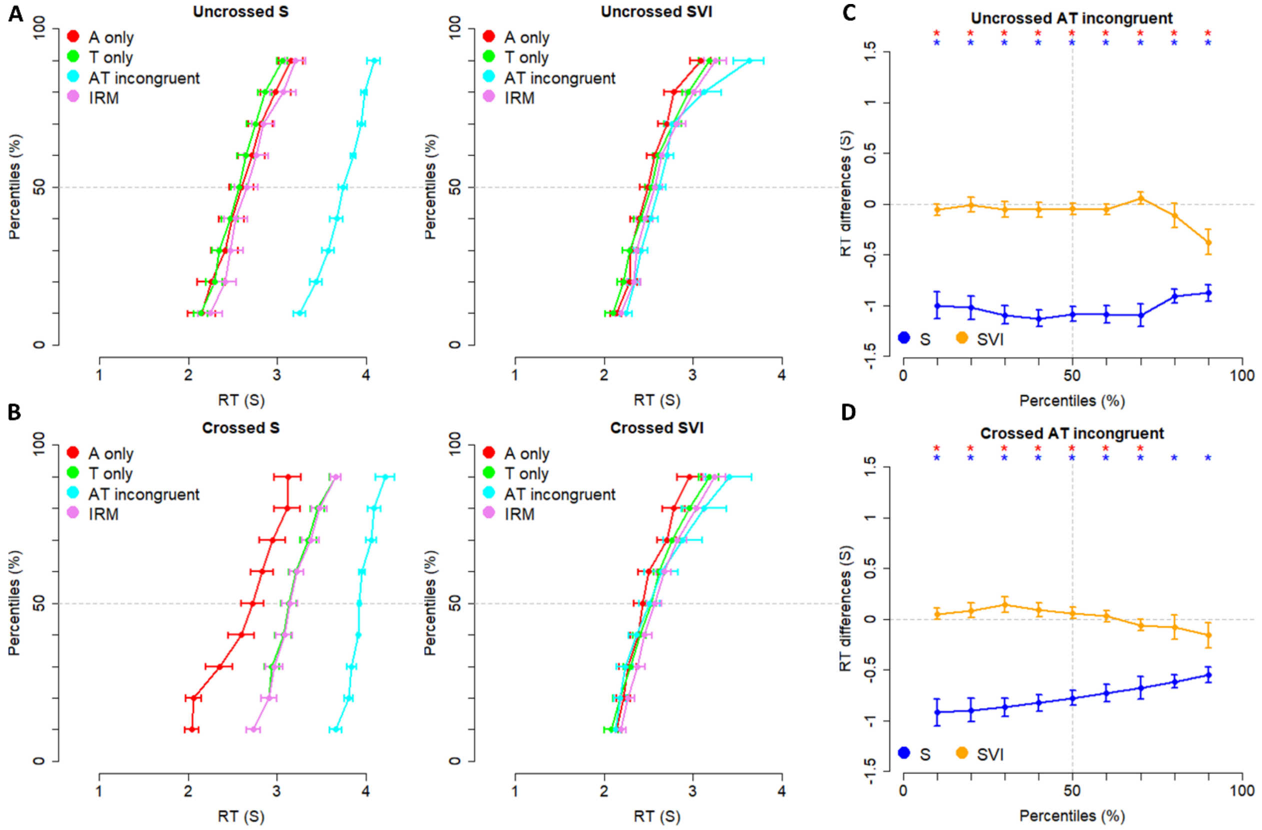

Figure S3. Comparisons of multisensory interference effects in Severely Visually Impaired (SVI) and Sighted (S) infants via comparisons of the extent of "inverted" redundancy gains. Related to Figure 3. An inverted race model is considered, testing reaction times in incongruent trials against the maximum of unimodal reaction times. This analysis tests if the presentation of spatially conflicting auditory and tactile cues slowed responses down significantly compared to the slowest motor reaction times in the summed cumulative distribution functions (CDFs) of the two unisensory conditions. A and B. For each posture (A: Uncrossed; B: Crossed), CDFs are presented for responses to Auditory only (red), Tactile only (green), Audiotactile incongruent (cyan) stimuli, and inverted RM prediction (IRM). C and D. Tests of inverse redundancy gains, for each Posture (C: Uncrossed; D: Crossed), Group (blue: S; orange: SVI), and percentile (10%-90%). Blue and yellow asterisks indicate inverse redundancy gains in the S and SVI infants, respectively ($p < .05$ after Bonferroni correction for $n = 9$ comparisons). Red asterisks indicate significant differences in inverse redundancy gains between S and SVI infants ($p < .05$ after Bonferroni correction for $n = 9$ comparisons). This analysis indicates that the sighted infants' RTs were slowed more in the audiotactile incongruent condition than could be expected by the slowest (longest) reaction times in the unisensory conditions. There was a significantly greater inverse redundancy gain in the sighted compared to the SVI group.

	Trial no.	Stimulus condition	Left hand	Right hand
**Blocks 1, 2** (Uncrossed-hands), & **4, 5** (Crossed-hands)	1	AT congruent	AT	
	2	AT congruent		AT
	3	AT incongruent	A	T
	4	AT congruent	AT	
	5	A only		A
	6	AT incongruent	T	A
	7	AT incongruent	A	T
	8	A only		A
	9	A only	A	
**Blocks 3** (Uncrossed-hands) & **6** (Crossed-hands)	1	T only	T	
	2	T only		T
	3	T only	T	
	4	T only	T	
	5	T only		T
	6	T only	T	

Table S1. Detailed order of trials presented to each infant participant. Related to Figure 1. There were 6 blocks of trials, with blocks 4, 5, and 6 a repetition of blocks 1, 2, and 3, but in a crossed hands posture. Stimulus conditions were: i) Auditory only trials ("A only"), where a single auditory stimulus was presented to either the left or the right hand, ii) Congruent audiotactile trials ("AT congruent"), where auditory and tactile stimuli were presented to a single hand simultaneously, iii) Incongruent Audiotactile trials ("AT incongruent"), where auditory and tactile stimuli were presented across separate hands simultaneously, and iv) Tactile only trials ("T only"), where a single tactile stimulus was presented to either the left or the right hand. The presentation of stimuli in left and right hands are detailed in the last two columns (A = Auditory stimulus, T = Tactile stimulus, AT = Audiotactile stimulus).

Group	Age (m)	Gender	Neuro-ophthalmological pathology	Grating acuity	Class of impairment
**Severely visually impaired (SVI)**	5	Male	Nystagmus and roving eye movements	1.7 cy/deg	SVI
	6	Male	Inherited retinal dystrophy (Leber's Congenital Amaurosis)	Sporadic light perception at very close distance	CB (complete)
	15	Female	Microphthalmia in left eye with bilateral ocular coloboma and catarct	2.40 cy/deg (light perception only in left eye)	SVI
	17	Female	Oculocutaneous albinism	4.7 cy/deg	MVI/SVI
	20	Male	Microphthalmia in right eye with bilateral large chorioretinal and iris coloboma	2.20 cy/deg	SVI
	27	Male	Bilateral optic nerve hypoplasia	Sporadic light perception at very close distance	CB (complete)
	28	Male	Bilateral optic nerve hypoplasia	1.3 cy/deg	SVI
	29	Female	Retinal detachment in bilateral stage 5 retinopathy of prematurity (ocular prosthesis in right eye)	Sporadic Low Vision; Teller Card perception at close distance	CB (Partial)
	32	Male	Inherited retinal dystrophy	4.7 cy/deg	MVI/SVI
	35	Female	Inherited retinal dystrophy (achromatopsia)	2.4 cy/deg	SVI
**Sighted (S)**	8	Male	-	-	-
	20	Male	-	-	-
	21	Female	-	-	-
	25	Male	-	-	-
	27	Female	-	-	-
	27	Male	-	-	-
	27	Female	-	-	-
	27	Female	-	-	-
	28	Female	-	-	-
	31	Male	-	-	-

Table S2. Details of the participants including clinical details of the visually impaired participants. Related to Figure 2. Participants' genders and age in months are presented. The data points in Figures. 2, S1 and S2 are presented in order of age in months. In the severely visually impaired (SVI) group neuro-ophthalmological pathologies, grating acuities (assessed via Teller acuity cards where possible) and class of impairment are reported (CB = Congenitally blind; MVI = Moderate visual impairment; SVI = Severe Visual Impairment).

Group	Stimulus condition	Posture	Total completed trials	Disagreement		Null		Analyzed			RT> 4 s
				Side	Modality	Both	None	Eye	Hand	Eye & hand	
Sighted (S)	A only	Uncrossed-hands	60	2	1	1	5	10	38	3	0
		Crossed-hands	48	3	2	1	5	11	22	4	0
	AT congruent	Uncrossed-hands	60	0	0	1	1	7	44	7	0
		Crossed-hands	57	0	0	0	2	10	38	7	0
	AT incongruent	Uncrossed-hands	60	1	0	3	3	15	32	6	6
		Crossed-hands	50	1	0	1	2	17	25	4	11
	T only	Uncrossed-hands	60	1	0	2	2	14	35	6	0
		Crossed-hands	60	0	0	1	3	10	42	4	0
Severely Visually Impaired (SVI)	A only	Uncrossed-hands	60	1	0	1	1	10	41	6	0
		Crossed-hands	47	0	1	1	1	12	27	5	0
	AT congruent	Uncrossed-hands	60	0	0	8	4	13	30	5	0
		Crossed-hands	53	0	0	3	6	8	33	3	0
	AT incongruent	Uncrossed-hands	60	1	0	3	3	10	40	3	5
		Crossed-hands	49	3	0	3	2	9	28	4	6
	T only	Uncrossed-hands	60	0	0	2	6	6	41	5	0
		Crossed-hands	60	0	2	6	2	10	38	2	0

Table S3. Details of trials completed and trials submitted to analyses across groups and conditions. Related to Figure 2. For each group, condition and posture, this table reports the number of: total completed trials; trials in which there was an inter-rater disagreement about side or modality (these were not included in analyses); trials which were coded as a null response due to a movement of both hands or an absence of motion within 8 seconds (these were not included in analyses; a marginally significant effect of group on the numbers of null trials was recorded, $t(156.6) = 1.973$, $p = .05$.); trials submitted to analysis, i.e. trials in which an orienting response was agreed between coders, subdivided across orienting responses of the eye (or head), of the hand, or both; trials where the response was made at a latency > 4 seconds (these were included in the analyses reported in the paper, but we determined that their exclusion did not modify the pattern of findings

Contrast list 1	Group	Orienting Direction (GLMM)			Reaction Time (LMM)		
		z	p	d	z	p	d
Uncrossed - Crossed	Sighted	3.712	<.001*	1.950	6.296	<.001*	2.882
	SVI	.173	.863	.093	.606	.553	.273

Contrast list 2	Posture	Orienting Direction (GLMM)			Reaction Time (LMM)		
		z	p	d	z	p	d
Sighted – SVI	Uncrossed	-.145	.885	-.078	.096	.924	.044
	Crossed	-.665	<.001*	-1.935	-5.643	<.001*	-2.565

Table S4. Results of the contrasts investigating tactile localization and body representation analyses for both generalised linear mixed models (GLMMs) applied to the direction of infants orienting responses (Left columns) and linear mixed models (LMMs) applied to reaction times (RTs; Right columns). Related to Figure 2. For the 2 way interaction of Group x Posture, the Bonferroni correction was applied considering contrasts of interest, i.e., for each level of one factor, the pairwise comparisons were made between all levels of the other factor. For each group (S/SVI) we compared postures (contrast list 1, in emmeans: list(pairwise~posture|group), n = 2 comparisons); for each posture we compared groups (contrast list 2, in emmeans: list(pairwise~group|posture), n = 2 comparisons). * = significant comparisons.

MULTISENSORY INTEGRATION MODELS

Contrast list 1	Group	Condition	Orienting Direction (GLMM) z	p	d	Reaction Time (LMM) z	p	d
Uncrossed - Crossed	Sighted	Auditory	.156	.876	.078	-.448	.656	-.205
	Blind	Auditory	-.026	.979	-.012	.678	.500	.306
	Sighted	Congruent	1.237	.216	1.519	2.688	.009*	1.230
	Blind	Congruent	-1.074	.283	-.823	.424	.672	.192
	Sighted	Tactile	4.358	<.001*	1.900	-9.828	<.001*	-4.498
	Blind	Tactile	-.088	.930	-.040	.095	.925	.043

Contrast list 2	Condition	Posture	Orienting Direction (GLMM) z	p	d	Reaction Time (LMM) z	p	d
Sighted – Visually impaired	Auditory	Uncrossed	.137	.891	.061	.472	.641	.453
	Congruent	Uncrossed	1.934	.053+	2.272	-2.877	.008*	-2.765
	Tactile	Uncrossed	.274	.784	.121	-.079	.938	-.076
	Auditory	Crossed	-.057	.954	-.029	1.003	.326	.964
	Congruent	Crossed	-.083	.934	-.070	-3.958	.001*	-3.804
	Tactile	Crossed	-4.096	<.001*	-1.819	4.647	<.001*	4.465

Contrast list 3	Posture	Group	Orienting Direction (GLMM) z	p	d	Reaction Time (LMM) z	p	d
Auditory - Congruent	Uncrossed	Sighted	-2.707	.020*	-3.087	14.726	<.001*	6.741
Auditory - Tactile	Uncrossed	Sighted	.016	1.000	.007	.594	1.000	.272
Congruent - Tactile	Uncrossed	Sighted	2.722	.019*	3.094	-14.132	<.001*	-6.469
Auditory - Congruent	Crossed	Sighted	-2.422	.046*	-1.645	17.861	<.001*	8.176
Auditory - Tactile	Crossed	Sighted	3.767	<.001*	1.829	-8.786	<.001*	-4.022
Congruent - Tactile	Crossed	Sighted	5.442	<.001*	3.474	-26.647	<.001*	-12.197
Auditory - Congruent	Uncrossed	Blind	-2.667	.052+	-1.895	7.806	<.001*	3.523

Auditory - Tactile	Uncrossed	Blind	.157	1.000	.067	-.570	1.000	-.257
Congruent - Tactile	Uncrossed	Blind	2.756	.054+	1.753	-8.375	<.001*	-3.780
Auditory - Congruent	Crossed	Blind	-2.350	.056+	-1.687	7.552	<.001*	3.408
Auditory - Tactile	Crossed	Blind	.083	1.000	.039	-1.153	.758	-.520
Congruent - Tactile	Crossed	Blind	2.427	.046*	1.726	-8.705	<.001*	-3.928

CROSSMODAL CONFLICT MODELS

			**Orienting Direction (GLMM)**			**Reaction Time (LMM)**		
**Contrast list 1**	**Group**	**Condition**	z	p	d	z	p	d
Uncrossed - Crossed	Sighted	Auditory	-.270	.787	-.059	-.355	.723	-.163
	Blind	Auditory	.442	.658	.089	.538	.592	.243
	Sighted	Incongruent	-2.626	.009*	-.530	-2.867	.005*	-1.312
	Blind	Incongruent	.837	.403	.175	1.533	.129	.692
	Sighted	Tactile	-7.286	<.001*	-1.383	-7.796	<.001*	-3.568
	Blind	Tactile	.070	.944	.014	.075	.940	.034

			**Orienting Direction (GLMM)**			**Reaction Time (LMM)**		
**Contrast list 2**	**Condition**	**Posture**	z	p	d	z	p	d
Sighted – Visually impaired	Auditory	Uncrossed	.512	.611	.146	.496	.623	.360
	Incongruent	Uncrossed	7.999	<.001*	2.291	8.113	<.001*	5.882
	Tactile	Uncrossed	.035	.972	.010	-.083	.934	-.060
	Auditory	Crossed	.956	.343	.294	1.055	.299	.765
	Incongruent	Crossed	9.932	<.001*	2.996	1.877	<.001*	7.886
	Tactile	Crossed	4.912	<.001*	1.407	4.886	<.001*	3.542

			**Orienting Direction (GLMM)**			**Reaction Time (LMM)**		
**Contrast list 3**	**Posture**	**Group**	z	p	d	z	p	d
Auditory - Incongruent	Uncrossed	Sighted	-13.200	<.001*	-2.598	-14.666	<.001*	-6.713

Auditory - Tactile	Uncrossed	Sighted	.306	1.000	.060	.472	1.000	.216
Incongruent - Tactile	Uncrossed	Sighted	13.783	<.001*	2.658	15.138	<.001*	6.929
Auditory - Incongruent	Crossed	Sighted	-13.864	<.001*	-3.069	-17.178	<.001*	-7.863
Auditory - Tactile	Crossed	Sighted	-5.965	<.001*	-1.265	-6.969	<.001*	-3.190
Incongruent - Tactile	Crossed	Sighted	9.035	<.001*	1.804	1.209	<.001*	4.673
Auditory - Incongruent	Uncrossed	Blind	-2.369	.054+	-.453	-1.638	.275	-.691
Auditory - Tactile	Uncrossed	Blind	-.398	1.000	-.076	-.452	1.000	-.204
Incongruent - Tactile	Uncrossed	Blind	1.925	.164	.377	1.687	.291	.687
Auditory - Incongruent	Crossed	Blind	-1.688	.276	-.367	-1.644	.313	-.742
Auditory - Tactile	Crossed	Blind	-.731	1.000	-.152	-.914	1.000	-.413
Incongruent - Tactile	Crossed	Blind	1.017	.928	.216	.729	1.000	.329

Table S5. Results of the contrasts carried out for multisensory integration analyses (top), and crossmodal conflict analyses (bottom) for both generalised linear mixed models (GLMMs) applied to the direction of infants orienting responses (Left columns) and linear mixed models (LMMs) applied to reaction times (RTs; Right columns). Related to Figure 2. For the 3 way interaction of Group x Posture x Condition, the Bonferroni correction was applied considering contrasts of interest, i.e., for each level of two factors, pairwise comparisons were made between all levels of the third. For each group and condition we compared postures (contrast lists 1, in emmeans: list(pairwise~posture|group*condition), n = 6 comparisons); for each condition and posture we compared groups (contrast lists 2, in emmeans: list(pairwise~group|condition*posture), n = 6 comparisons); for each posture and group we compared conditions (contrast lists 3, in emmeans: list(pairwise~condition|posture*group), n = 12 comparisons). * = significant comparisons (p<.05), while + = trends (p<.09).
